# Atypical Onset and Imaging Characteristics in Non-demyelinating Myelin Oligodendrocyte Glycoprotein Antibody-Associated Encephalitis: A Case Report and Literature Review

**DOI:** 10.7759/cureus.70722

**Published:** 2024-10-02

**Authors:** Neha A Patel, Camila Narvaez Caicedo, Sylvia V Jimenez, Courtney K Mack, Xiang Fang

**Affiliations:** 1 Neurology, John Sealy School of Medicine, University of Texas Medical Branch, Galveston, USA; 2 Neurology, University of Texas Medical Branch, Galveston, USA

**Keywords:** acute encephalitis syndrome, mini mental score examination, myelin-oligodendrocyte glycoprotein antibody disease, negative magnetic resonance imaging, non demyelination

## Abstract

Myelin oligodendrocyte glycoprotein antibody-associated disease (MOGAD) is typically associated with central nervous system inflammation and demyelination in younger populations; however, rare cases of isolated encephalitis in the elderly have been reported. This article presents the case of an 80-year-old male with mild cognitive impairment who developed progressive cognitive decline and a focal seizure. Despite negative imaging findings, elevated serum MOG antibody titers and pleocytosis in cerebrospinal fluid (CSF) analysis suggested MOG antibody encephalitis. Following treatment with intravenous immunoglobulin (IVIg), the patient showed significant cognitive improvement, and MOG antibody titers decreased, indicating a positive response. This case highlights the diverse clinical spectrum of MOGAD, demonstrating its potential to cause encephalitis in elderly individuals, even in the absence of typical imaging findings, and emphasizes the importance of promptly recognizing MOG antibody encephalitis to reduce associated mortality and morbidity.

## Introduction

Myelin oligodendrocyte glycoprotein antibody-associated disease (MOGAD) encompasses a broad spectrum of autoimmune disorders characterized by central nervous system (CNS) inflammation and demyelination [1]. MOGAD phenotypes vary and have been proven to follow an age-dependent pattern, as children typically present with acute disseminated encephalomyelitis, while adults present with optic neuritis or transverse myelitis [2]. Regardless of phenotypic presentation, cerebrospinal fluid (CSF) analysis and imaging findings share similar characteristics due to the shared pathophysiology of demyelination. Limited studies have described rare cases of image-negative MOGAD isolated encephalitis in children and adults, with even fewer cases reported in elderly individuals [3-5]. We report a case of an 80-year-old man with imaging-negative MOG antibody encephalitis without focal neurological deficits.

## Case presentation

An 80-year-old African American man with pre-existing mild cognitive dysfunction of unspecified etiology, who was able to carry out all activities of daily living, presented to the emergency department (ED) with a seven-day history of progressive confusion, somnolence, right-sided weakness, retro-orbital headache, and difficulty walking and performing fine motor skills. Before ED arrival, the patient’s wife described an approximately 60-second episode of unresponsiveness, forced head deviation to the right, and right-hand tremor suggestive of a focal seizure. His past medical history included bilateral lacunar cerebellar infarcts without residual deficits, coronary artery disease status post coronary artery bypass graft, paroxysmal atrial fibrillation on anticoagulation, uncontrolled type 2 diabetes mellitus, stage 3 chronic kidney disease (CKD), hypertension, and hyperlipidemia. 

Four weeks prior, the patient was admitted for retro-orbital headache and transient blurry vision in the right eye in the setting of elevated erythrocyte sedimentation rate (85 mm/h). He was started empirically on steroids to treat possible giant cell arteritis (GCA) but later discontinued after the temporal artery biopsy was unrevealing. The social history was relevant for a 30-pack-year history of tobacco use, but he quit 44 years ago and reported no current substance use. 

On initial presentation, the patient was febrile (38.2°C) with otherwise normal vital signs, lethargic, and oriented only to the person. He was unable to provide an accurate history or follow commands. Strength was 4+/5 strength in all extremities, with normal deep tendon reflexes except 1+ Achilles tendon bilaterally. There were no signs of meningeal irritation or inflammation. Initial complete blood count, complete metabolic panel, and urinalysis were benign. A lumbar puncture was not obtained at the time due to the risk of bleeding in the setting of anticoagulation therapy. An electroencephalogram revealed moderate diffuse slowing but no epileptiform discharges. Inflammatory markers, such as antinuclear antibody, high-sensitivity C-reactive protein, and erythrocyte sedimentation rate, were elevated at 1:160 (reference interval <1:40), 1.83 mg/dL (reference interval <0.74 mg/dL), and 63 mm/h (reference interval 2-30 mm/h), respectively.

The patient received empiric antibiotics and antiviral treatment for suspected infectious encephalitis, but his symptoms failed to improve. By day 7 of admission, his mental status continued to deteriorate. A lumbar puncture was performed in light of the patient’s lack of clinical improvement. CSF analysis revealed mild pleocytosis with a white blood cell count of 6 (reference interval 0-5/μL), lymphocytic dominance (80% lymphocytes), elevated protein at 147 mg/dL (reference interval 15.0-45.0 mg/dL), glucose of 80 mg/dL (reference interval 50-80 mg/dL), and two oligoclonal bands (reference interval 0-1). Bacterial, mycobacterial, and fungal cultures showed no growth, and polymerase chain reaction (PCR) tests for *Cytomegalovirus*, *Enterovirus*, *Escherichia coli K1*, *Haemophilus influenzae*, herpes simplex virus 1, herpes simplex virus 2, human herpesvirus 6, human parechovirus, *Listeria monocytogenes*, *Neisseria meningitidis,* *Streptococcus agalactiae, Streptococcus pneumoniae, *Varicella-Zoster virus (VZV), and *Cryptococcus neoformans *and* C. gattii *were negative. The autoimmune encephalitis panel resulted 12 days after admission. The serum MOG antibody immunoglobulin G (IgG) titer, assessed using a semi-quantitative cell-based indirect fluorescent antibody assay, was strongly positive at 1:320 (normal <1:10). Aquaporin-4 (AQP4), N-methyl-D-aspartate (NMDA), contactin-associated protein 2 (CASPR2), leucine-rich glioma-inactivated (LG1) protein, α-amino-3-hydroxy-5-methyl-4-isoxazolepropionic acid (AMPA) receptor, gamma-aminobutyric acid receptor types A and B (GABA-BR, GABA-AR), dipeptidyl aminopeptidase-like protein 6, anti-IgLON family member 5 (IgLON5), metabotropic glutamate receptor 1 (mGluR1), and voltage-gated potassium channel (VGKC) antibodies were negative in serum. 

**Figure 1:**
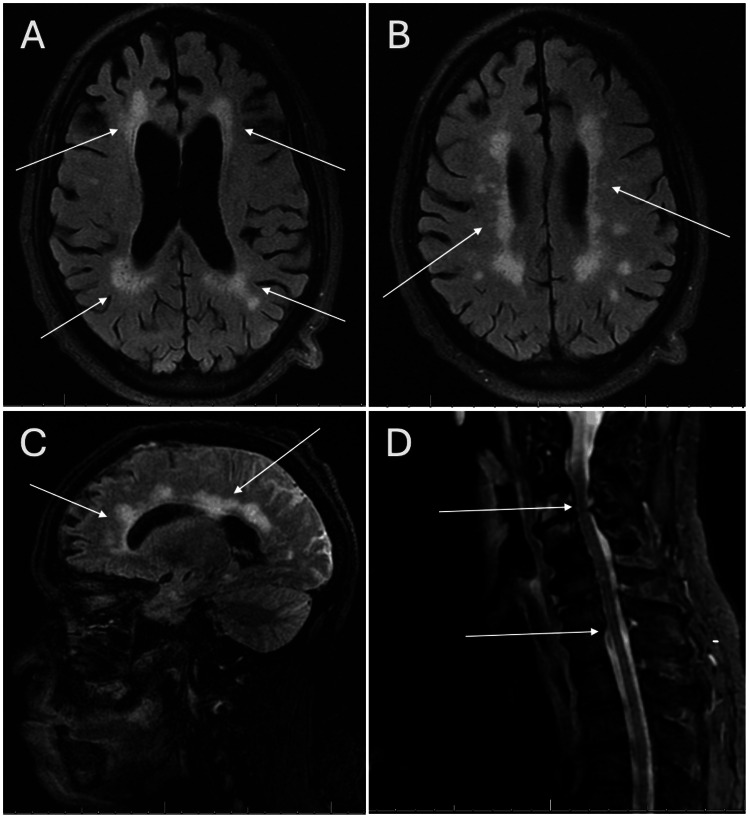
Brain MRI scan with and without contrast (A-C) showing non-enhancing confluent bilateral periventricular and deep white matter FLAIR hyperintensities likely related to sequelae of chronic ischemic small vessel disease, in addition to mild global cerebral volume loss with sulcal widening and ex vacuo dilation of the ventricles. These lesions were not enhanced. Cervical spine (D) MRI with and without contrast shows multilevel spondylitis changes without abnormal cervical cord signal alteration or post-contrast enhancement. MRI: magnetic resonance imaging; FLAIR: fluid-attenuated inversion recovery.

Magnetic resonance imaging (MRI) of the brain and cervical spine was obtained (Figure 1). Brain imaging revealed periventricular and deep white matter T2/FLAIR (fluid-attenuated inversion recovery) hyperintensities suggestive of chronic microvascular disease, and additional foci of parenchymal gradient blooming signal in the left temporal lobe and bilateral cerebellar hemispheres were suggestive of chronic infarcts. Contrast images were deferred due to a history of stage 3 CKD. MRI of the orbit without contrast was limited due to motion artifact, and a non-contrast MRI of the cervical spine was negative for any acute inflammatory changes.

The patient was initiated on 2 g/kg of intravenous immunoglobulin (IVIg) over five days, and treatment efficacy was assessed using daily Mini-Mental State Examination (MMSE). The pre-treatment MMSE score was 11/30 but rose to 21/30 one day after completion of the IVIg therapy. He was discharged with a return to baseline mental status and a one-month steroid taper. The patient was followed up in the outpatient neurology clinic with fluctuating cognitive impairments. Six months after discharge, repeat serum MOG antibody titer levels improved to 1:80. 

## Discussion

Neuroinflammatory diseases of the CNS form a diverse spectrum of immunological origins. Continued research has provided the identification of CNS antibodies, such as myelin oligodendrocyte glycoprotein, and expanded the clinician’s insight into disease etiologies [1]. MOGAD is now recognized as a distinct disease entity with an immunopathogenesis that differs from both multiple sclerosis (MS) and AQP4-IgG-positive neuromyelitis optica spectrum disorder (NMOSD) [1]. Unfortunately, MOGAD’s clinical course and variant phenotypes are not well elucidated in the literature in contrast to MS and NMOSD, and its clinical and radiological similarities to other demyelinating disorders often lead to misdiagnosis. So far, the most common way to diagnose MOGAD is with the presence of serum MOG-IgG1 antibodies in conjunction with radiologic-positive demyelinating lesions and clinical findings.

The evaluation and diagnosis of MOGAD involve serum testing, CSF analysis, and CNS imaging. CSF analysis reveals pleocytosis, a nonspecific inflammatory marker, and CNS MRI commonly reveals cortical swelling and T2/FLAIR hyperintensities with gadolinium enhancement, an indirect marker of CNS inflammation [6]. These lesions have poorly defined margins commonly found in the white matter of the brain, spine, or orbit. Adjacent subcortical T2/FLAIR hypodensities have been seen in patients with encephalitis and are hypothesized to be a landmark of the disease phenotype [6-8]. Although radiographic imaging for our patient demonstrated diffuse white matter changes, the patient’s multiple underlying cerebrovascular risk factors, as well as the location and symmetry of those lesions, indicate that these were not active demyelinating lesions but rather chronic microvascular changes.

In March 2023, a proposed diagnostic criteria for MOGAD was published in Lancet Neurology that may help to stratify future diagnoses of MOGAD [9]. Diagnosis requires fulfillment of three criteria: (1) A core demyelinating event, such as optic neuritis, myelitis, acute disseminated encephalomyelitis (ADEM), cerebral unifocal or multifocal deficits, brainstem or cerebellar deficits, or cerebral cortical encephalitis with the presence of seizures; (2) positive MOG IgG test using a cell-based assay with supporting clinical or radiographic findings; and (3) the exclusion of other better diagnoses, including MS [9]. In our case, the presence of significantly elevated MOG antibody titer (1:320) renders the likelihood of a false positive result to be minimal and therefore supports our diagnosis of MOG antibody encephalitis, despite a lack of typical radiographic findings. This leads us to conclude that our patient demonstrated a phenotype that challenges said criteria by having no radiographic findings consistent with demyelinating disease.

While relevant literature on image-negative MOG antibody encephalitis is limited, similar reports on the existence of this specific disease phenotype have been published. Macaron and Ontaneda present the case of a 57-year-old man with a one-week history of subacute urinary retention, which progressed to rapidly worsening symmetric paraparesis and reduced sensation in both lower extremities up to the umbilicus. Despite significant clinical symptoms, CNS MRI conducted 48 hours after symptom onset was normal, except for two non-enhancing supratentorial T2 abnormalities. MOG-IgG1 testing on a fluorescence-activated cell sorting assay was positive with a titer of 1:10,000. The exclusion of other etiologies, therefore, confirmed the diagnosis of MOGAD encephalitis. Intravenous methylprednisolone improved his symptoms, such that MOG-IgG titers decreased to 1:100 at five months post-discharge [4]. Our case not only highlights a case of image-negative MOGAD encephalitis similar to that of Macaron and Ontaneda but presents without the presence of focal neurological deficits, other than the aforementioned impairments to mental status and cognition. Additionally, our patient demonstrated clinical improvement in response to steroid therapy, thus fitting with the clinical picture of autoimmune encephalitis.

One report by Fujimori et al. postulated that the existence of image-negative MOG antibody encephalitis may occur if a patient has had a recent history of steroid therapy [10]. In our case, our patient had a prior history of suspected GCA and received a two-week steroid taper with remission of symptoms. In retrospect, given the patient’s lack of biopsy findings consistent with GCA, it could be suggested that his initial symptoms of headache and blurred vision were manifestations of MOGAD before diagnosis. It is also possible that our patient’s treatment with systemic steroids may have prevented any CNS demyelination that could be detected on MRI. Other possible hypotheses suggest that the presence of image-negative findings in MOGAD may be related to variable timing of radiographic imaging (MRI is conducted too early or late to capture transient inflammation or demyelination), limited MRI sensitivity to transient inflammation of demyelination in gray matter tracts, or limited MRI sensitivity to detect microscopic changes in the oligodendrocyte cytoskeleton caused by the MOG antibody [3]. While it is unclear if some or all of these hypotheses played a part in our patient’s image-negative presentation, it does not negate the presence of MOGAD and its ability to cause long-lasting and permanent neurological impairment, particularly in the elderly population.

## Conclusions

While multiple reports of image-positive MOG antibody encephalitis have allowed clinicians to understand the disease and its implications, the presentation and prognosis of image-negative MOG antibody encephalitis are not as well elucidated in the current literature. We seek to rectify this by reporting on a rare phenotype of this disease that challenges the standard clinical presentation. Clinicians ought to maintain a high degree of clinical suspicion for MOG antibody encephalitis even in the absence of typical radiographic abnormalities, as prompt recognition and treatment initiation are essential to mitigate adverse sequalae associated with this condition. Future research should focus on developing comprehensive case series, identifying reliable biomarkers, and conducting therapeutic trials to enhance understanding and management of this challenging autoimmune disorder. Through these efforts, we can improve the quality of care for patients with MOGAD and reduce the burden of this debilitating disease.
